# Correlation between synthesis of α_2_-macroglobulin as acute phase protein and degree of hepatopathy in rats

**DOI:** 10.1186/s42826-019-0014-0

**Published:** 2019-08-17

**Authors:** Reina Ito, Takashi Kuribayashi

**Affiliations:** Laboratory of Immunology, School of Life and Environmental Science, Azabu University, Fuchinobe 1-17-71, Chuo-ku, Sagamihara, Kanagawa 252-5201 Japan

**Keywords:** α2M, Galactosamine, Hepatopathy, Cut-off value, Rats

## Abstract

The degree of hepatopathy affecting the synthesis of α_2_-macroglobulin (α2M) as an acute phase protein in rats was investigated. Hepatopathy was induced in Sprague-Dawley rats by intravenous administration of galactosamine at a dose of 30 mg/kg for 7 days. Inflammation was induced by intramuscular injection of turpentine oil at a dose of 2 mL/kg. Blood was collected before turpentine oil injection and at 24, 48, 72 and 96 h after injection. Serum concentrations of α2M were measured by enzyme-linked immunosorbent assay. Mean values of aspartate aminotransferase (AST) and alanine aminotransferase (ALT) in rats administered galactosamine were significantly higher than in controls. Mean values of body weight and total protein were significantly lower than in controls. Serum concentrations of α2M in the galactosamine group were significantly lower than in controls. Kinetic parameters, area under the concentration-time curve (AUC^0–96^) and maximum serum concentration (Cmax), were significantly lower than in controls. The cut-off value for detecting the effects on synthesis of α2M in liver was 46.9 mgˑh/mL. Seven rats (77.8%) were assessed for decreases in the synthesis of α2M due to hepatopathy. Two rats showed no influence on the synthesis of α2M, despite administration of galactosamine. AST and ALT in these two rats were ≤ 285 and ≤ 174 U/L, respectively. In conclusion, synthesis of α2M in rats is evidently suppressed in the severe stages of hepatopathy.

## Introduction

α_2_-macroglobulin (α2M) is a protein inhibitor with broad specificity in humans [1–4]. For example, chymase, a mast cell serine protease, was inhibited by α2M [5]. Although it is not an acute phase protein in humans [6–8] it is a typical acute phase protein in rats [9–13]. Serum concentrations of α2M show increased sensitivity than α_1_-acid glycoprotein in rats in response to inflammatory stimulation [10]. Thus, α2M is a useful inflammatory marker in rats [12, 13]. α2M is synthesized in the liver, and production is decreased with hepatic impairment [14, 18]. Many candidate drug substances are reported to induce hepatopathy [15–17]. Evaluation of the degree of inflammation using serum concentrations of α2M may therefore give inaccurate results when assessing candidate substances that induce hepatopathy. Serum biochemical parameters, such as AST and ALT, show abnormally high values in rats with hepatopathy, while serum concentrations of α2M are lower than in normal rats [18]. However, the correlation between the extent of liver function failure and the decrease in α2M synthesis in the liver has not been clarified. Moreover, it has not been investigated how much liver damage affects the synthesis of α2M. Thus, the cut-off value for reductions in the serum concentration of α2M in rats with hepatopathy was determined from receiver-operating characteristic (ROC) curve analysis. Moreover, correlations between serum biochemical parameters and α2M were investigated in order to clarify how much liver damage affects the synthesis of α2M.

## Material and methods

### Animals

Twenty male Sprague-Dawley rats (age, 6 weeks) were purchased from CLEA Japan, Inc. (Tokyo, Japan). Rats were divided into two groups; the galactosamine group, and the control group. Rats were kept in isolators at a temperature of 23 ± 2 °C on a 12/12 dark/light cycle (6:00–18:00). Rats were fed MF (Oriental Yeast Co., Ltd., Tokyo, Japan) and were allowed free access to water.

### Animal experimental designs

**Fig. 1:**
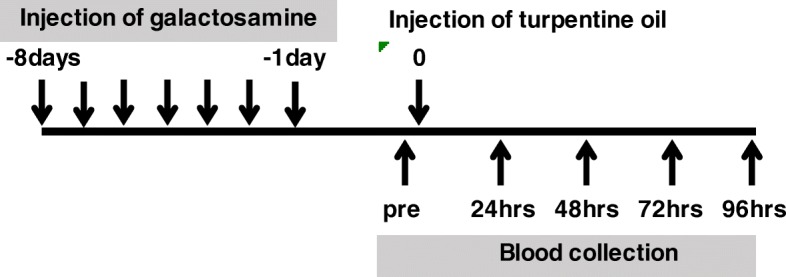
Experimental protocol to evaluate the degree of hepatopathy affecting the synthesis of α2-macroglobulin (α2M) in rats. Galacatosamine was injected into rats at 30 mg/kg once a day for 7 days. Turpentine oil was injected at 2 ml/kg. Blood was collected before injection of turpentine oil, and at 24, 48, 72 and 96 h after injection

The animal experimental protocol of this study is shown in Fig. 1. Hepatopathy was induced in 10 rats by intravenous injection of D(+)-Galactosamine Hydrochloride (Wako Pure Chemical Industries, Ltd., Osaka, Japan) at 300 mg/kg (5 mL/kg) daily for 7 days. The other ten rats (control group) were intravenously injected with sterilized saline. Turpentine oil is known to induce acute inflammation and has been used to induce acute inflammation in rats [19]. In this study, turpentine oil (Wako Pure Chemical Industries, Ltd.) was thus used to induce acute inflammation by intramuscular injection at 2.0 mL/kg body weight the day after the end of galactosamine administration. Blood (0.3 mL) was collected from the venae cervicalis superficialis under anesthesia by inhalation of isoflurane (Wako Pure Chemical Industries, Ltd.) at pre-injection of turpentine oil, and at 24, 48, 72 and 96 h after injection. Serum was obtained by centrifugation (1600×g, 15 min) and was stored at − 80 °C until use. All experiments were approved by the Institutional Review Board of Azabu University (approval No. 170324–1).

### Measurement of serum concentrations of α2M

Serum concentrations of α2M were measured by enzyme-linked immunosorbent assay (ELISA) according to the procedure described by Honjo et al. [20].

### Serum biochemical analysis

Aspartate aminotransferase (AST) and alanine aminotransferase (ALT) were measured by the ultraviolet method. Total protein (TP) was measured by the Biuret method.

### Statistics

Data were analyzed using GraphPad Prism 7.0 software (La Jolla, CA, USA). All values are expressed as means ± SEM. Area under the concentration-time curve (AUC) for α2M was calculated according to the trapezoidal rule [21, 22]. Variations in serum concentrations of α2M, AST, ALT and TP were assessed using unpaired Student’s *t*-test. *P*-values of < 0.05 was considered to be significant. Cut-off values of α2M for detecting hepatopathy were determined from ROC curve analysis.

## Results

**Fig. 2:**
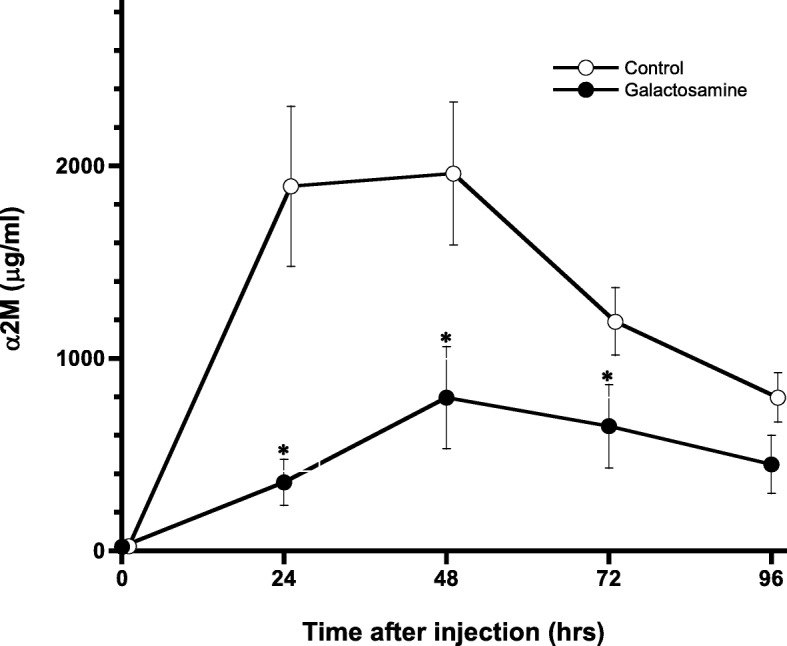
Changes in serum concentrations of α_2_-macroglobulin (α2M) in rats intravenously injected with galactosamine at 30 mg/kg once a day for 7 days. Data are means ± SEM (galactosamine; *n* = 9, control; *n* = 10). Differences were compared using unpaired Student’s *t*-test. *Significantly different from controls (*p* < 0.05)

The serum biochemical analysis results are shown in Table 1. Unfortunately, 1 rat in the galactosamine group died at 48 h after turpentine oil injection, due to the adverse effects of galactosamine. The mean values of AST and ALT in the galactosamine group were significantly higher than in the control group. Body weight and TP in the galactosamine group were significant lower than in control group. Changes in serum concentrations of α2M in the hepatopathy and control groups are shown in Fig. 2. The kinetic parameters of α2M are shown in Table 2. Mean serum concentrations of α2M at 24, 48 and 72 h after injection of turpentine oil in the control group were significantly lower than in the galactosamine group. Mean maximum serum concentration (Cmax) and AUC^0–96^ in the control group were significantly lower than in the galactosamine group.

**Table 1:** Serum biochemical analysis and body weight in rats intravenously injected with galactosamine at 30 mg/kg once a day for 7 days

Substances	Body weight (g)	AST (U/l)	ALT (U/l)	TP (g/dl)
Galactosamine	217* ± 6	1327* ± 332	934* ± 232	4.9* ± 0.3
Control	243 ± 6	74 ± 2	28 ± 2	5.7 ± 0.1

Data are represented mean ± SEM. Differences were compered using unpaired Student’s t-test. *Significantly different from control (*p* < 0.05)

**Table 2:** Kinetic parameters of a2-macroglobulin in rats intravenously injected with galactosamine at 30 mg/kg for 7 days

Substances	Cmax (mg/ml)	Cmax/pre	AUC^0–96^	(mg-hr/ml)
Galactosamine	799.5* ± 215.9	34.5* ± 11.5	44.9*	± 10.1
Control	2038.5 ± 397.1	85.3 ± 8.6	111.6	± 15.0

Data are represented as mean ± SEM. Differences were compared using the unpaired Student’s t-test. *Significantly differences from control (*p* < 0.05)

**Fig. 3:**
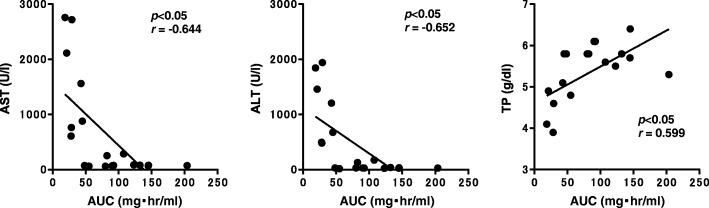
Correlation between area under the blood concentration-time curve (AUC^0–96^) of α_2_-macroglobulin and aspartate aminotransferase (AST), alanine aminotransferase (ALT) and total protein (TP) in rats intravenously injected with galactosamine at 30 mg/kg once a day for 7 days. *P* value and coefficient of correlation are shown

**Fig. 4:**
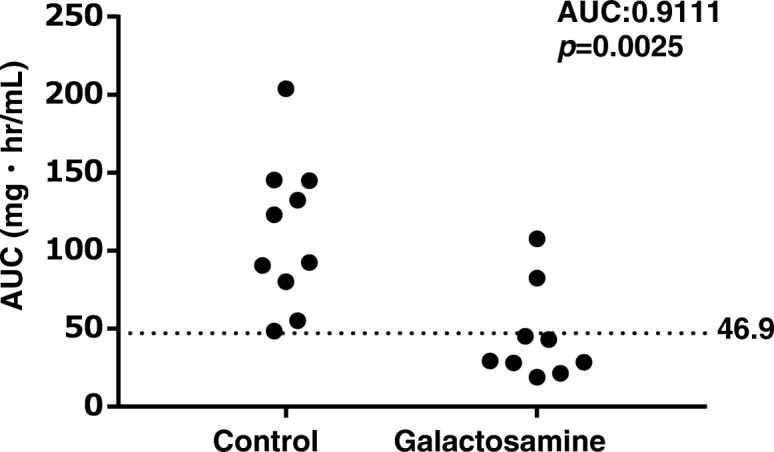
Dot plot showing the distribution of area under the blood concentration-time curve (AUC^0–96^) of α_2_-macroglobulin in rats intravenously injected with galactosamine at 30 mg/kg once a day for 7 days. The dotted line indicates the cut-off value determined from the receiver-operating characteristic (ROC) curve analysis. Cut-off value was 46.9 mgˑh/mL

**Fig. 5:**
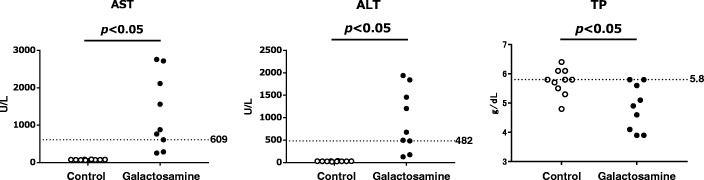
Dot plot showing the distribution of aspartate aminotransferase (AST), alanine aminotransferase (ALT) and total protein (TP) in rats intravenously injected with galactosamine at 30 mg/kg once a day for 7 days. Dotted line indicated the lowest value in rats considered to be showing altered synthesis of α_2_-macroglobulin based on the cut-off value of AUC^0–96^

The correlations between AUC^0–96^ and AST, ALT or TP are shown in Fig. 3. Significant negative correlations were observed between AUC^0–96^, and AST and ALT (AST: *r* = − 0.644, *p* < 0.05; ALT: *r* = − 0.652, *p* < 0.05). A significant positive correlation was observed between AUC^0–96^ and TP (*r* = 0.589, *p* < 0.05). Individual data, AUC^0–96^, AST, ALT and TP in the galactosamine and control groups are shown in Figs. 4 and 5. The cut-off value for AUC^0–96^ to detect hepatopathy was 46.9 mgˑh/mL by ROC analysis. Seven rats (77.8, 95%CI:0.78–1.05%) in the galactosamine group were assessed for decreased synthesis of α2M in liver (Fig. 4).

## Discussion

We evaluated how the hepatopathy induced by galactosamine affected the synthesis of α2M in rats. Inflammation was induced by injection of turpentine oil in this study. The effects of hepatopathy on synthesis of α2M in rats could be evaluated after single administration of turpentine oil [18]. Thus, single injection of turpentine oil was not considered to have influenced the synthesis of α2M, and turpentine oil was used to induce inflammation in this study. Significant differences between the galactosamine group and controls were observed in AST, ALT and TP. Administration of galactosamine was therefore considered to have induced hepatopathy in this study. Certainly, significant differences were observed in serum concentrations of α2M, AUC^0–96^ and Cmax between the galactosamine group and controls, suggesting that synthesis of α2M changed in rats after hepatopathy was induced by administration of galactosamine. Moreover, a synthetic decrease in α2M was also possible based on the significant negative correlation between AUC^0–96^ and AST and ALT.

Individual data were then evaluated to clarify the degree that hepatopathy affects the synthesis of α2M. Seven rats were judged to have shown changes in the synthesis of the α2M based on the cut-off value of AUC^0–96^. AST and ALT levels in these seven rats were more than 609 and 482 U/mL, respectively. Galactosamine is known to induce hepatopathy in experimental animals [23, 24]. Hepatopathy model rats are generated by administration of galactosamine in many studies [23–25]. AST and ALT in rats administered a single dose of galactosamine at 1100 mg/kg are reported to be 100.86 and 121.57 U/mL, respectively [26]. AST and ALT in rats administered galactosamine at dose of 800 mg/kg are reported to be 96 and 199 U/L, respectively [27]. AST and ALT showed higher values in this study than in hepatopathy model rats reported previously. On the other hand, AST and ALT in the two rats that showed no effect on the synthesis of α2M were less than or equal to 285 and 174 U/L, respectively. From these results, the synthesis of α2M was considered to be inhibited in severe hepatopathy stages. Estimation of α2M as an inflammatory marker will therefore be need to be carefully evaluated in non-clinical studies, particularly toxicological studies that use high dosages or evaluate substances that induce severe hepatopathy.

## Conclusions

The synthesis of α2M in rat liver was largely sustained until severe hepatopathy. However, caution is required when evaluating the degree of inflammation of substances that induce severe liver damage using α2M in non-clinical studies.

## Data Availability

All data generated or analyzed during this study are included in this submitted manuscript.

## Abbreviations

ALT: Alanine aminotransferase
AST: Aspartate aminotransferase
AUC: Area under the concentration-time curve
Cmax: Maximum serum concentration
ELISA: Enzyme-linked immunosorbent assay
ROC: Receiver-operating characteristic
TP: Total protein
α2M: α_2_-macroglobulin
